# Investigating *Mycoplasma wenyonii* and *Candidatus* Mycoplasma haematobovis coinfection patterns in cattle from southwestern France reveals strain-specific traits

**DOI:** 10.1186/s13567-026-01821-y

**Published:** 2026-08-03

**Authors:** Chloé Saada, Renaud Maillard, Claire Pelletier, Marie-Claude Hygonenq, Hortensia Robert, Christine Citti, Laurent-Xavier Nouvel

**Affiliations:** 1 ENVT, INRAE, IHAP, Univ Toulouse, 23 chemin des Capelles, 19 Cedex, 31076 Toulouse, France; 2 Laboratoire Départemental d’Analyses de l’Ain, Bourg-en-Bresse, France

**Keywords:** *Mycoplasma wenyonii*, *Candidatus* Mycoplasma haematobovis, hemoplasma, coinfections, cattle, France

## Abstract

Hemotropic mycoplasmas (hemoplasmas) infecting cattle were first detected in France in 2019 during a clinical outbreak, but their prevalence both at the species and strain levels remains unknown. This study therefore aimed to determine the prevalence of hemoplasmas and their coinfections, and to assess the within and between herd diversity in five fully sampled dairy herds. Detection was performed using 16S rRNA quantitative polymerase chain reaction (qPCR), as well as three strain-specific *rnpB* qPCR assays targeting *Candidatus* Mycoplasma haematobovis (CMh, formerly *Ca*. M. haemobos), *Mycoplasma wenyonii* strain Mexico (Mex), and *Mycoplasma wenyonii* strain Massachusetts (Mass). A total of 1011 cattle were sampled, of which 88.7% (897/1011) tested positive by 16S rRNA qPCR. Detection rates for CMh, Mex, and Mass were 67.7% (684/1011), 65.9% (666/1011), and 19.8% (200/1011), respectively. Concordance between 16S rRNA qPCR and strain-specific *rnpB* qPCR was 92% (931/1011). Coinfections were detected in 67.8% (565/833) of 16S PCR-positive animals, with the CMh + Mex combination being the most frequent. The Mass strain was predominantly found in association with the two other hemoplasmas. For all four PCR assays, hemoplasma positivity was significantly associated with both herd and animal age in the five fully sampled dairy herds. Further studies are needed to elucidate transmission routes and pathogenicity mechanisms of hemoplasmas.

**Supplementary Information:**

The online version contains supplementary material available at 10.1186/s13567-026-01821-y.

## Introduction

Hemotropic mycoplasmas, also referred to as hemoplasmas, are bacteria belonging to the *Mycoplasma* genus that infect the red blood cells of humans and animals. In cattle, *Mycoplasma wenyonii* (Mw) was the first hemoplasma species to be described, initially classified as *Eperythrozoon wenyonii* [1] before being reclassified within the *Mycoplasma* genus following taxonomic revisions based on 16S rRNA sequence analysis [2]. The complete genome sequence of the *M. wenyonii* strain Massachusetts (Mass) was obtained in 2012 [3], which enabled the development of molecular detection methods on the basis of targets other than rRNA genes [4]. Subsequently, a study reported the draft genome sequence of another bovine hemoplasma species, *Candidatus* Mycoplasma haematobovis (CMh) strain INIFAP01, isolated from the blood of sick cattle [5]. The same research group later published the draft genome of another *M. wenyonii* isolate, strain INIFAP02 or Mexico (Mex) [6].

Transmission of hemotropic mycoplasmas is suspected to occur via blood-sucking insects and ticks, which are proposed as potential vectors [7, 8]. However, scientific demonstration of transmission to cattle via arthropods for *M. wenyonii* or *Ca.* M. haematobovis remains lacking [7]. Vertical transmission, though documented, is considered rare [8], and transmission through fomites contaminated with blood are suspected but has yet to be confirmed [9, 10]. Since their discovery, clinical manifestations associated with hemoplasma infection have been inconsistent. Early experimental infections in splenectomized calves primarily resulted in mild anemia and hyperthermia [1]. More recent studies have detected hemoplasmas in both clinically ill and apparently healthy cattle, with infection potentially leading to a range of symptoms, including: anemia, abortion, infertility, delayed estrus in cows, and in severe cases, icterus with hemoglobinuria as well as edema and drop in milk production [4, 11–13]. Chronically infected cows may also exhibit subclinical productivity losses such as reduced weigh at birth and reduced milk production [14]. Despite these observations, clinical outbreaks remain rare compared with the high global prevalence of hemoplasma infections [15], with recent studies reporting detection rates ranging from 50.8% to 88.39% in cattle and buffalo [16, 17]. The factors determining the shift from asymptomatic carriage to clinical disease remain poorly understood. Because hemoplasmas are non-cultivable, most knowledge relies on epidemiological studies, which have highlighted significant gaps in our understanding of their pathogenesis. One prevailing hypothesis is that coinfections among hemoplasma species may influence disease expression. However, previous research has primarily focused on species-level interactions, rather than strain-level dynamics.

To address these gaps, the objectives of this study are threefold: (1) to document the occurrence and genetic diversity of hemoplasma species and strains in cattle from southwestern France, including the potential identification of novel, undescribed species or strains; (2) to characterize coinfections at the strain level, providing finer-resolution insights; (3) to assess factors associated with each hemoplasma detection in a subset of five fully sampled dairy herds.

## Materials and methods

### Animals and samples

A convenience sample of 42 cattle herds was collected between December 2020 and January 2025. The study population included 33 beef herds (*n* = 400 cows) and 9 dairy herds (*n* = 611 cows), for a total of 1011 cows from 18 different breeds. Sampling was conducted with the owners’ consent and in compliance with national animal health regulations. Depending on farmer consent, between 1 and 167 cows per herd were sampled on the same day, which in some cases corresponded to the entire herd. All cows were eligible for inclusion, regardless of their breed, parity, production level, stage of lactation, reproductive status, or clinical expression. Whole blood samples (5 mL) were collected from the coccygeal vein into ethylenediaminetetraacetic acid (EDTA) tubes within the framework of clinical activities and French national bovine prophylaxis program. The latest is mandatory for all herds and targets animals over 2 years of age that thus represented the majority of sampled animals (867 out of 1011 cows).

For each animal, metadata were collected, including sampling date, date of birth, sex, age, breed, clinical expression, production system (dairy or beef cattle), and department (French administrative unit). Clinical expression was defined as any abnormal posture or behavior observed by the farmer or veterinarian at the time of sampling. Additionally, climatic data associated with each herd’s location were gathered from the Copernicus Climate Data Store [18] to account for potential environmental influences and vector-borne transmission of hemotropic mycoplasma infections. These included sampling season, climatic type (defined by geographic location), and annual mean daily temperature (°C), relative humidity (%), precipitation (mm), and wind speed (m/s).

Geographic coordinates (latitude/longitude) for each herd were derived from postal addresses, and climatic variables were assigned using the nearest 30 km-resolution grid point from the ERA5 reanalysis dataset [19]. Herds were classified as either Mediterranean (latitude < 46°N, longitude > 3°E; Köppen-Geiger Csa/Csb: hot, dry summers, mild winters) or semi-oceanic climate (outside the Mediterranean zone; Cfb: moderate temperatures, evenly distributed precipitation). Herds near climatic boundaries were classified according to the dominant climate of their department, as defined by the Köppen–Geiger classification [20].

Blood samples were transported at 4 °C to the National Veterinary School of Toulouse and stored at −20 °C until DNA extraction.

### Laboratory analyses

#### DNA extraction

Total DNA was extracted from 100 µL of whole EDTA blood using a commercial magnetic bead-based extraction kit (Mag Fast ID.VET, IDvet, France) following the manufacturer’s instructions. Briefly, the extraction protocol consisted of cell lysis and DNA binding, washing with a final wash containing 80% ethanol, drying, and elution. Purified DNA was eluted in 80 µL of the kit’s elution buffer. To monitor DNA extraction efficiency and assess the absence of PCR inhibitors, a control quantitative polymerase chain reaction (qPCR) assay targeting the mitochondrial 12S rRNA gene was performed on each sample, as described elsewhere [21].

#### Polymerase chain reaction (PCR) assays

Extracted DNA was used in a real-time PCR assay using SYBR Green chemistry targeting the 16S rRNA gene with universal primers for all hemoplasmas of the *Mycoplasma* genus, as previously described [22, 23]. Additionally, three qPCR assays targeting the *rnpB* gene were performed to detect *Candidatus* Mycoplasma haematobovis (CMh), *Mycoplasma wenyonii* strain Massachusetts (Mass), and *Mycoplasma wenyonii* strain Mexico (Mex). The assays followed established protocols [24], modified for TaqMan PCR with primers and probes adapted from publicly available genomic sequences. Primer specifications and PCR cycling conditions are described in Table 1. PCR reactions were carried out using a Light Cycler 96 (Roche, Basel, Switzerland). Each 25 µL reaction mixture contained 2 µL of the sample, 0.4 µM of each primer, 1 × PCR buffer supplemented with MgSO_4_ (New England Biolabs, Évry, France), 0.2 mM each dNTP, and 0.25 µL of Taq DNA polymerase (New England Biolabs). Negative controls (nuclease-free water) were included in each PCR run to monitor for contamination. Positive controls consisted in quantified plasmid standards for each PCR, which were used to validate amplification performance. Results were considered negative for cycle thresholds over 38, 36, 36, and 35 for 16S rRNA, *rnpB* CMh, *rnpB* Mex, and *rnpB* Mass, respectively. These thresholds were defined on the basis of a combination of standard curve performance, signal reproducibility, and consistency with both positive and negative controls.

**Table 1:** **qPCR conditions used in this study**

Designation	Target	Locus	Primers	TaqMan probe	Fragment size (bp)	Annealing temp (°C)	Extension time (s)	No. cycles
16S rRNA	Hemoplasma	16S rRNA gene	For – 5’- ACGAAAGTCTGATGGAGCAATA -3’ Rev – 5’- ACGCCCAATAAATCCGRATAAT -3’	None (SYBR Green qPCR)	197	60	30	35
CMh	*Candidatus* Mycoplasma haematobovis	*rnpB* gene	For -5’-TGATGACGCGGGTATATAAAAAATAA-3’ Rev – 5’ TCTCTGTGGCACTGGTCATCAC-3’	5’-FAM-CCCGACACCTTTTG-MGB-3’	92	58	30	30
Mex	*Mycoplasma wenyonii* Mexico	*rnpB* gene	For – 5’-AGTCTGAGATGACTATAGTGATTGTGTGAG-3’ Rev – 5’-TTTAGAAAGGTTCTCCGCCATC-3’	5’-FAM-AACTCAACGGCTAGTCTGACTAG-MGB-3’	92	58	30	30
Mass	*Mycoplasma wenyonii* Massachusetts	*rnpB* gene	For – 5’-AGTCTGAGATGACTGTAGTGTTTGTGTAAG-3’ Rev – 5’-TTAGAAAGGTTCTCCGCCATCA-3’	5’-FAM-AACTTAACGGCTAGTAATAGACT-MGB-3’	92	58	30	30

#### PCR sensitivity and specificity, plasmid-based controls, and sequence analysis

Positive controls consisted of quantified plasmid standards. PCR amplicons were purified using the QIAquick PCR Purification Kit (Qiagen, Courtaboeuf, France) and cloned into the pGEM-T Easy vector (Promega, Charbonnières-les-Bains, France).

After transformation into *Escherichia coli* DH5α competent cells (Invitrogen/Life Technologies, Courtaboeuf, France), plasmids were purified with the QIAprep Spin Miniprep Kit (Qiagen). Insertion of target DNA fragments was confirmed by PCR and Sanger sequencing, performed at the Eurofins facility (Köln, Germany). Sequence quality and assembly were assessed using Chromas Lite software, and sequence identity was confirmed by Basic Local Alignment Search Tool (BLAST) analysis against the NCBI non-redundant nucleotide database (nr/nt) restricted to *Mollicutes*. Plasmid DNA concentration was quantified using a Qubit fluorometer (Thermo Fisher Scientific, Villebon-sur-Yvette, France). PCR sensitivity was evaluated using serial dilutions of purified plasmids and positive field samples, with each dilution tested in triplicate. The limits of detection were determined to be 5.8 × 10^3^ DNA genome equivalents per mL of whole blood for 16S rRNA assay, 2.3 × 10^5^ genome equivalents per mL of whole blood for *rnpB* CMh target, and 5.36 × 10^2^ and 3.8 × 10^5^ genome equivalents per mL of whole blood for *rnpB* Mex and *rnpB* Mass, respectively. Amplification specificity was assessed through melt curve analysis, which consistently showed a single, well-defined peak, supporting the absence of significant nonspecific amplification. The specificity of the 16S rRNA PCR was previously evaluated elsewhere [4], and a subset of amplicons underwent Sanger sequencing; the obtained sequences were confirmed to correspond to hemotropic mycoplasmas. Upon testing, the specific *rnpB* PCR assays showed no cross-reactivity in between species (CMh, Mw) or strains (Mw-Mass, Mw-Mex) and with other bacteria (*Mycoplasma agalactiae, M. arginini, M. bovis, M. bovirhinis, M. gallisepticum*, and *Anaplasma phagocytophilum*). Each assay successfully amplified samples containing the targeted hemoplasma, including when mixed with one or two other hemoplasma strains. No amplification was observed for samples containing only nontarget hemoplasma, alone or in mixtures. Additionally, the assays were evaluated on a subset of samples with known infection status, previously characterized by Sanger sequencing of 16S qPCR amplicons, further supporting the specificity of the *rnpB* assays (data not shown).

### Data management and statistics

Data coding and management were performed using MS Excel. Age at sampling was categorized into four classes: 0–1 year, 2 years, 3 years, and 4 years and more to account for key management-related transition and physiological statuses in dairy herds (0–1 year: youngstock, 2 years: around first calving; 3 years: primiparous cow; 4 years and more: multiparous cow).

Animals with missing data for one or more required variables were excluded prior to analysis. This was exclusively due to the lack or absence of reported clinical expression, as only six cows were suspected of infection. As this number was insufficient for relevant analysis, these animals were removed from the dataset. All animals included in the dataset had complete results for all four PCR assays (16S, and three *rnpB*-specific assays). Analyses were performed using R software (version 4.2.2; RStudio), with statistical significance set at *p* < 0.05. The unit of analysis was the individual animal. Categorical variables were analyzed using the chi-squared test or Fisher’s exact test (for *n* < 5), while nonparametric data were analyzed using the Wilcoxon test (with Bonferroni correction where applicable). Univariate mixed-effects logistic regression model (fitted using the glmer function in R) were used to assess the association between age class and hemoplasma detection (binary outcome: 0 = negative, 1 = positive). The analysis included 488 cows from 5 fully sampled herds. Given the nonlinear relationship between age and positivity, age was categorized in previously mentioned age classes and included as a fixed effect, with the 0–1 year class as the reference. Herd was included as a random intercept to account for clustering within herds. Model fit was assessed using DHARMa residuals, with no evidence of overdispersion (*p* > 0.05).

## Results

### High overall hemoplasma detection with species and strain specific variations

Hemoplasma detection was performed on blood samples from 1011 cows using 4 qPCR assays: 1 targeting the 16S rRNA gene of all hemoplasma species including *Mycoplasma wenyonii* and *Candidatus* Mycoplasma haematobovis, and 3 targeting the *rnpB* gene to specifically amplify *Candidatus* Mycoplasma haematobovis (CMh), *Mycoplasma wenyonii* strain Mexico (Mex), and *Mycoplasma wenyonii* strain Massachusetts (Mass).

Results presented in Table 2 indicate high hemoplasma detection rate in this study since 88.7% (897/1011) were positive using 16S rRNA qPCR assay. Among these, 92.9% (833/897) also tested positive with at least one *rnpB* specific assay and further considered as “concordant results.” Whether the remaining 7.1% (64/897, later referred to as “16S only”) reflect detection of hemoplasmas other than those studied or differences in PCR sensitivities and specificities remains unknown.

**Table 2:** **Prevalence of hemoplasma species detection among the 1011 sampled cattle**

Specific qPCR results		16S positive	16S negative	Total positive (%) *
Hemoplasmas		897	114	1011
*Candidatus* Mycoplasma haematobovis	Positive	673	11	67.7%
*Mycoplasma wenyonii* Strain Mexico	Positive	655	11	65.9%
*Mycoplasma wenyonii* Strain Massachussetts	Positive	197	3	19.8%
CMh and Mex and Mass	Negative	64 (1)	98 (2)	
CMh or Mex or Mass	Positive	833 (3)	16 (4)	

^*^Total exceeds 1011 because of codetections; (1) discordant result: “16S only”; (2) concordant result: no detection; (3) concordant result: “16S and at least one specific PCR positivity”; (4) discordant result: “specific PCR only”

A small proportion of cattle, 9.7% (98/1011), were PCR-negative with all four assays. Surprisingly, 1.6% (16/1011) of the animals turned out to be negative with the 16S rRNA qPCR assay but positive with at least one of the specific PCR (later referred to as “specific PCR only”). More specifically, five cows were positive with “CMh” alone, five with “Mex” alone, three with “CMh + Mex,” and three with “CMh + Mex + Mass.”

Finally, “discordant result” were observed with “16S-only” results accounting for 6.3% (64/1011) of total samples and “specific PCR only,” representing 1.6% (16/1011) of total samples. Overall, the 16S and *rnpB*-specific PCR assays yielded concordant results in 92% (931/1011) of total samples. The prevalence of detected hemoplasma varied among species and strains. Whereas CMh and Mex were the most frequently detected hemoplasmas with 67.7% (684/1011) and 65.9% (666/1011) of positive samples, respectively, Mass was the least prevalent, detected only in 19.8% (200/1011) of samples. Given these high detection rates, codetections were suspected to be frequent and were further categorized as follows: no detection (ND), single-detection (SD), dual-detection (DD), and triple-detection (TD).

### Differential hemoplasma loads in species and strains, with 16S rRNA loads exhibiting differences depending on specific PCR results

**Figure 1:**
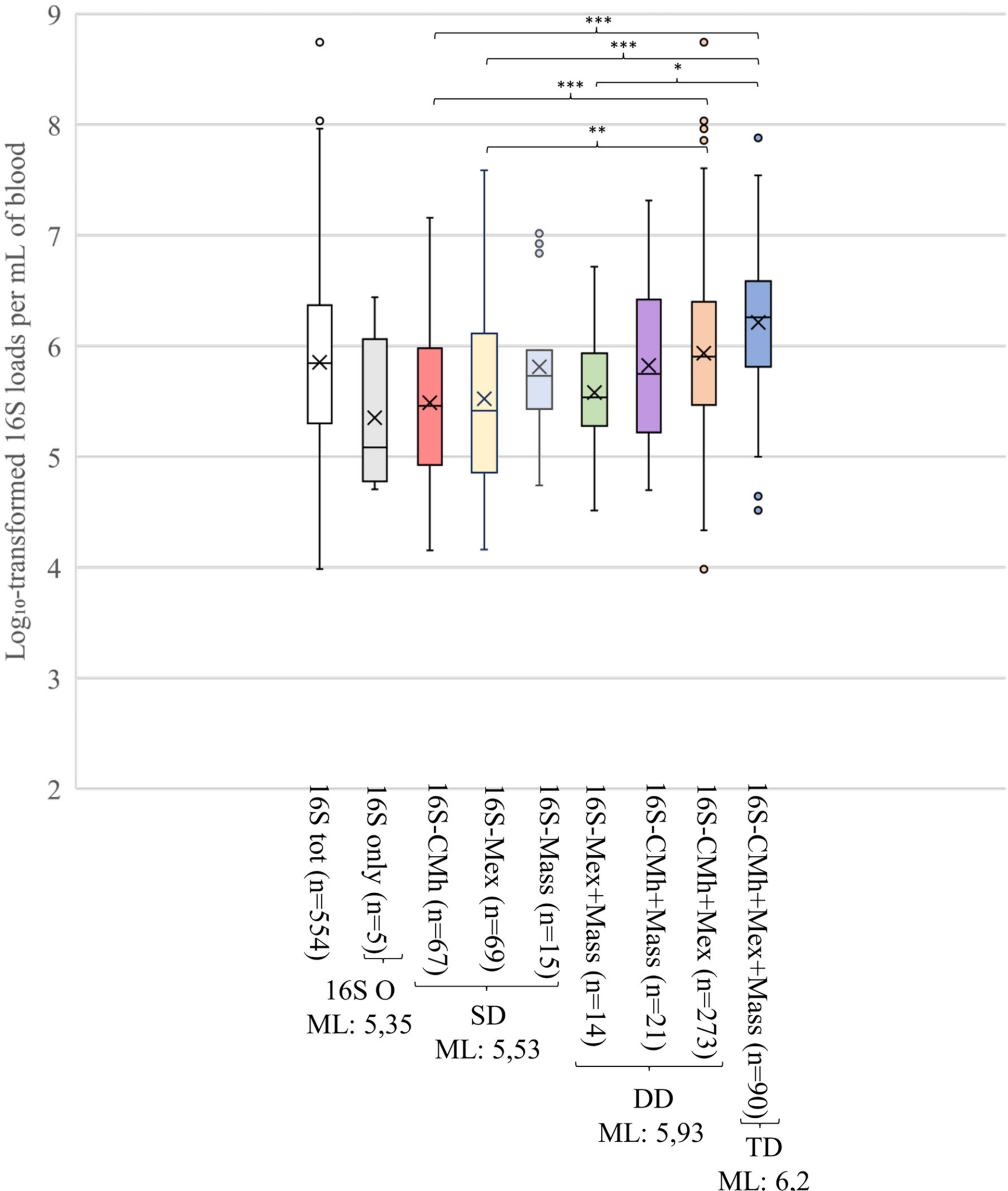
**Log₁₀-transformed hemoplasma loads per mL of blood for each PCR (A) and 16S rRNA PCR loads according to hemoplasma detection categories (B) in successfully quantified samples.** 16S O: “16S only”; *SD* single detection, *DD* dual detection, *TD* triple detection, *ML* mean load per mL of blood. Statistical significance: *** *p* < 0.001, ** *p* < 0.01, * *p* < 0.05 (Wilcoxon test with Bonferroni correction). Mex log₁₀-transformed loads above 9 (*n* = 23, ranging from 9.04 to 31.57) were excluded from this figure for clarity but were included in all statistical analyses (additional file 3).

To better address the discrepancies observed above, for 16S-positive samples that were negative with specific-qPCR assays or vice versa, the hemoplasma loads were estimated in all 1011 samples (Figure 1). Quantification was performed as described in the Material and Methods section and was successful in 61.8% (554/897), 52.6% (360/684), 61.6% (410/666), and 57% (114/200) of positive samples for 16S-, CMh-, Mex- and Mass-PCRs, respectively (Figure 1-A). Unsuccessful quantification was related to low DNA template concentration in a subset of samples, which resulted in Ct values within the detection range but below the quantification threshold of the assay.

When compared, mean log₁₀-transformed hemoplasma loads per mL of blood were the highest for 16S and Mex (5.85 ± 0.76 and 5.92 ± 2.02 copies/mL blood, respectively, Figure 1-A). Hemoplasma loads (Figure 1-B) appeared to be correlated with the number of detected species and strains per sample: TD (6.21 ± 0.64 copies) presented significantly higher loads than DD (5.93 ± 0.73 copies, *p* = 0.02) and SD (5.53 ± 0.76 copies, *p* < 0.01), and DD presented significant higher loads than SD (*p* < 0.01).

This trend was further supported by the analysis of hemoplasma loads stratified by detailed codetection type. Both TD (6.21 ± 0.64 copies) and DD − CMh + Mex (5.9 ± 0.71 copies) presented significantly higher loads than SD − CMh (5.48 ± 0.73 copies; *p* < 0.01 for both comparisons) and SD − Mex (5.52 ± 0.79 copies; *p* < 0.01 for both comparisons). Most of the codetections involving Mass were not significant, likely due to limited statistical power (small sample size: *n* = 114 successfully quantified Mass-positive samples, Figure 1B). However, the partial success of the quantification assay may introduce a selection bias, as successfully quantified samples are likely enriched for higher bacterial loads.

Mean log₁₀-transformed hemoplasma loads varied between species and strains with CMh = 5.09 ± 0.97 copies, Mex = 5.92 ± 2.02 copies, and Mass = 5.01 ± 1.23 copies, with Mex showing the widest distribution (Figure 1A). Elevated bacterial loads (mean log₁₀-transformed load > 8) accounted for 0.5% (3/554), 2.5% (9/360), 7.8% (32/410), and 0% (0/114) of quantified 16S, CMh, Mex, and Mass PCRs, respectively. However, direct comparison of absolute load values between the 16S rRNA and *rnpB* assays should be interpreted with caution, as these assays differ in target gene copy number, amplification efficiency, and overall sensitivity. Among the 32 samples with elevated Mex loads, 24 originated from a single herd (Herd 2, Cantal), which had a history of clinical cases compatible with hemoplasma infections, suggesting that herd-related factors may influence hemoplasma infection dynamics. The remaining eight samples were distributed across three other herds in Haute-Garonne.

Only 5 out of 64 “16S-only” samples were successfully quantified, yielding a lower mean bacterial load (5.35 ± 0.71 copies) than any other infection category among 16S-quantified samples. This supports the hypothesis that the 16S PCR assay may be more sensitive than some species-specific qPCR, enabling detection of low bacterial loads of one or more hemoplasma species/strains—sufficient to trigger 16S PCR positivity but below the detection threshold of specific qPCRs. This interpretation is further supported by the fact that, upon repetition of the 16S PCR, only 7 of the 64 “16S-only” samples retested positive: 2 were identified as *Mycoplasma wenyonii*, 2 as *Candidatus* Mycoplasma haematobovis, and 3 remained unidentified after amplicon sequencing.

Comparison of mean log₁₀-transformed CMh and Mass PCR loads by detailed codetection type revealed no significant differences (additional file 1). However, variations in Mex bacterial loads depending on the detected hemoplasma species or strains suggest that coinfection may influence the detection process, as the bacterial load of Mex was significantly higher in “16S − CMh + Mex + Mass” category (6.17 ± 1.69 copies) and the “16S − CMh + Mex” category (6.02 ± 2.3 copies) compared with the “16S -Mex” category (5.1 ± 1.1 copies; *p* = 0.042 and *p* = 0.026, respectively; additional file 2). Further exploration of codetections and herd-related factors is therefore warranted.

### Coinfections: high detection rates and strain-specific patterns within species

If hemoplasma detection is frequent among the samples tested in this study, codetection also occurred quite often. The prevalence and patterns of codetection were analyzed in the 92% (931/1011) of samples with concordant results between 16S and *rnpB* specific assays (Table 3).

**Table 3:** **Hemoplasma species and strain codetections**

Detection type	Species and strains	Number of samples	Percentage
No detection (ND)		98	10.5%
Single detection (SD)	CMh	127	13.6%
	Mex	119	12.8%
	Mass	22	2.4%
Dual detection (DD)	CMh + Mex	390	41.9%
	CMh + Mass	29	3.1%
	Mex + Mass	19	2.1%
Triple detection (TD)	CMh + Mex + Mass	127	13.6%
Total		931	100%

Only 28.8% (268/931) of cows harbored a single detection, while an even smaller proportion (10.5%) of samples yield no detection. Dual detections were the most common, accounting for 47% (438/931) of samples, predominantly involving the CMh + Mex combination. Triple detections were relatively rare (13.6%) as were codetections involving Mass, reflecting its low overall prevalence. Taken together, codetections were highly prevalent, occurring in 67.8% (565/833) of animals that tested PCR positive and had concordant results. The distribution of codetection patterns differed markedly among positive samples for each hemoplasma species and strain (*n* = 673 for CMh, *n* = 655 for Mex, and *n* = 197 for Mass). CMh and Mex exhibited strikingly similar profiles: single detection occurred in 18.9% and 18.2% of cases, respectively, but dual detections was predominant, especially with one another (57.9% and 59.5%). Their association with Mass was rare (4.3% and 2.9%), while both appeared in 18.9% and 19.4% of the cases in triple detections. In contrast, Mass displayed a distinct pattern: it was predominantly found in triple detections (64.5%), and only occasionally codetected with CMh (14.7%) or Mex (9.6%), or as single (11.2%).

**Figure 2:**
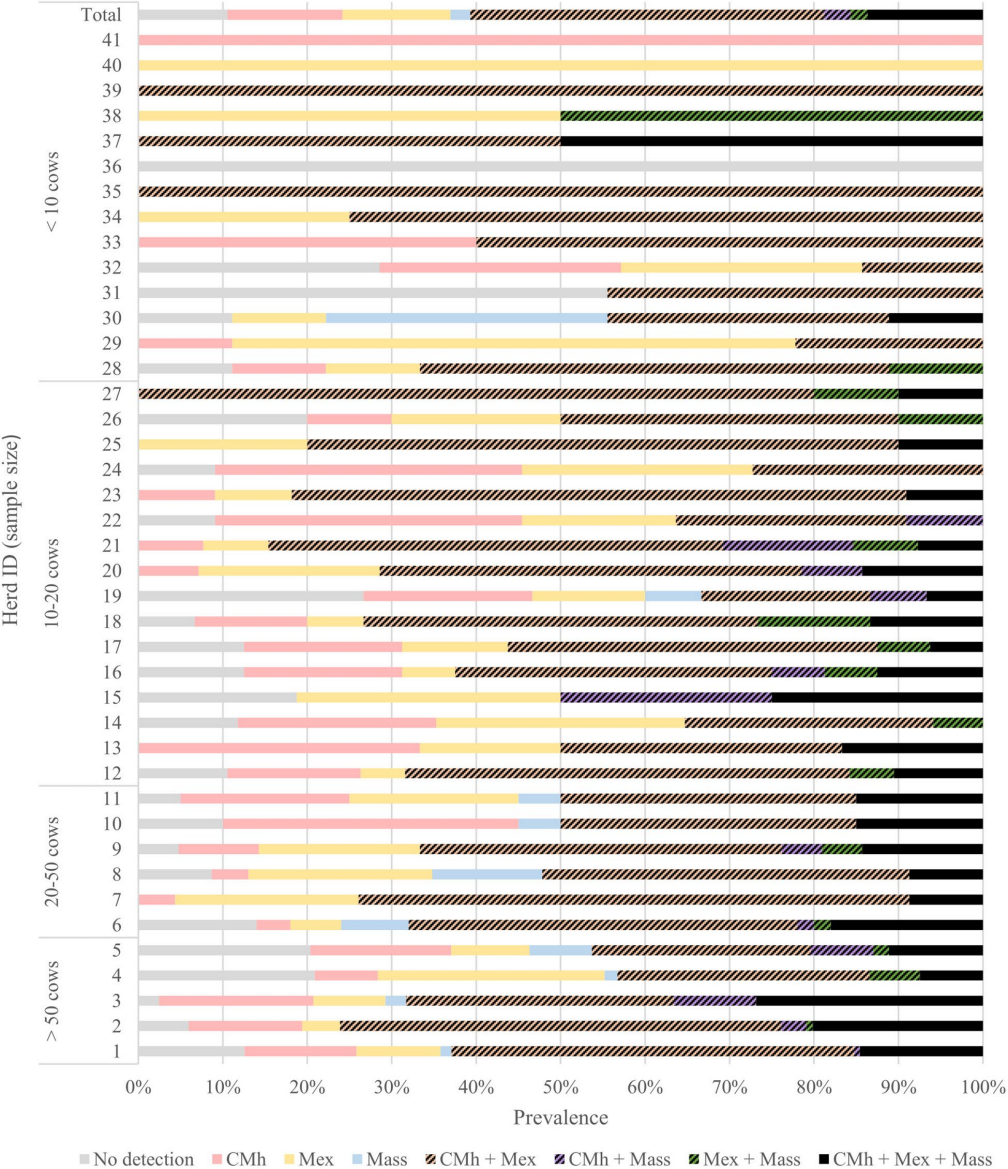
**Codetection patterns within herds (*n* = 931 cows from 41 herds). **Colored solid bars represent single detections; hatched bars represent dual detections; black solid bars represent triple detections. For > 50 sampled cows, herds were entirely sampled (herd 1: *n* = 151; herd 2: *n* = 134; herd 3: *n* = 82; herd 4: *n* = 67; herd 5: *n* = 54). Herd 42 (*n* = 1) is not represented because its only sample was part of the discordant results (“16S only”).

Analysis of herd-level coinfection patterns (Figure 2) revealed substantial variability in the prevalence of ND, SD, DD, and TD among herds, though small sample sizes sometimes obscured these patterns. For herds with at least 10 samples (herds 1–27, *n* = 867 cows), mean within-herd detection patterns were characterized by 9% ND cows/herd (range 0–26.7%), 31.1% SD cows/herd (range 0–63.6%), 48.7% DD cows/herd (range 25–90%), and 11.2% TD cows/herd (range 0–26.8%). These marked differences between herds suggest the influence of location-specific risk factors, potentially including climatic conditions and suspected vector-borne transmission dynamics of hemoplasma. However, some of the herd results may be biased by the inclusion of the concordant results only. Further investigations are therefore required to explain the observed between-herd variations.

### Hemoplasma detection prevalence and patterns across herds and age groups

To deepen the understanding of hemoplasma epidemiology, herd-related variables were analyzed using concordant results of 488 animals across 5 fully sampled herds (additional file 4).

Herds 2 and 3 (Cantal, semi-oceanic climate) exhibited the highest 16S (94–97.6%) and CMh (86.6–88.8%) detection rates, while herds 4 and 5 (Ardèche, Mediterranean climate) showed lower 16S (79.1–79.2%) and CMh PCR positive results (44.8–61.1%). Mass positivity peaked in herd 3 (39%), characterized by the highest annual mean daily precipitation, relative humidity, greater pasture access, and organic management. Herd 5 also showed elevated Mass detection rates (27.8%), suggesting that grazing during lactation (unique to herds 3 and 5) may be of influence. Herds 4 and 5 (sampled in summer) exhibited higher SD and lower TD proportions compared with herds 1–3 (sampled in spring/winter, Figure 2).

Although the above observations are purely descriptive, they may suggest potential seasonal fluctuations in hemoplasma prevalence, possibly driven by variations in vector activity or host susceptibility and between herd variability. Detection rates varied significantly by herd for all PCR targets (*p* < 0.01 for 16S, CMh, Mex, and Mass; chi-squared test). Age distribution across herds appeared to be a major factor underlying these variations, as evidenced by the univariate logistic regression analyses presented in Table 4. When considering all herds together, animals aged 2 years were more likely to test positive for 16S, CMh, Mex, and Mass compared with those aged 0–1 year. This pattern was also observed for animals aged 3 years compared with the 0–1 year reference group for 16S, CMh, and Mex. However, these results should be interpreted within the context of the five fully sampled herds and some estimates exhibited wide confidence intervals.

**Table 4:** **Odds ratios for PCRs positivity by age category in five dairy cattle herds**

PCR	Age category	OR	SE	95% CI	*p*-Value
16S	*Intercept (0–1 year)*	*2.73*	*1.01*	*[1.33; 5.65]*	*0.007*
	2 years	43.13	43.90	[5.86; 317.72]	** < 0.001**
	3 years	15.48	11.5	[3.6; 66.52]	** < 0.001**
	4+ years	3.63	1.23	[1.86; 7.06]	** < 0.001**
CMh	*Intercept (0–1 year)*	*1.90*	*0.84*	*[0.79; 4.54]*	*0.149*
	2 years	12.07	6.12	[4.47; 32.62]	** < 0.001**
	3 years	3.25	1.26	[1.52; 6.97]	**0.0024**
	4+ years	0.95	0.26	[0.55; 1.62]	0.838
Mex	*Intercept (0–1 year)*	*1.11*	*0.25*	*[0.72; 1.71]*	*0.646*
	2 years	7.06	2.54	[3.49; 14.28]	** < 0.001**
	3 years	3.83	1.35	[1.92; 7.63]	** < 0.001**
	4+ years	1.57	0.39	[0.96; 2.54]	0.069
Mass	*Intercept (0–1 year)*	*0.23*	*0.07*	*[0.13; 0.43]*	< *0.001*
	2 years	1.94	0.63	[1.02; 3.66]	**0.043**
	3 years	1.35	0.50	[0.65; 2.8]	0.417
	4+ years	1.23	0.38	[0.67; 2.23]	0.506

Results from mixed-effects logistic regression models with age as a fixed effect and herd as a random intercept. The reference category is 0–1 year. *OR* odds ratio, *SE* standard error, *95% CI* 95% confidence interval. Significant *p*-values (< 0.05) are in bold

When analyzed separately for each PCR and herd, the results were interesting yet inconsistent across PCRs and herds. Age had a significant effect on 16S positivity in herds 1, 4, and 5 (*p* < 0.01, Fisher’s exact test for all herds). For CMh positivity, age had a significant effect in herds 1 and 4 (*p* < 0.01, Fisher’s exact test and chi-squared test, respectively). For Mex positivity, age had a significant effect in herds 1, 2, 4, and 5 (*p* < 0.10, chi-squared test for herd 1; *p* = 0.04, *p* = 0.02, and *p* = 0.03, Fisher’s exact test for herds 2, 4, and 5, respectively). For Mass positivity, age had a significant effect in herds 2 and 3 (*p* < 0.01, chi-squared test for herd 2; *p* = 0.02, Fisher’s exact test for herd 3; additional file 5). This apparent disparity may be related to differences in grazing regimens and potential vector exposure among herds.

Herd 2, which exhibited the highest milk yields, also presented a history of clinical cases (edema of the hind limbs, milk loss, fever) with *Mycoplasma wenyonii* detection.

Within-herd analyses confirmed no significant differences in milk production between infected and non-infected cows at any lactation stage for any of the hemoplasma species when considering cows sampled before the onset of or during the first 3 months of lactation (Wilcoxon test, *p* > 0.05 for herds 1, 2, 3, and 4, all PCR, at all lactation stage except one; additional file 6).

Overall, location, climate, and management practices may by drivers of hemoplasma detection. To fully elucidate these dynamics, further investigations should include detailed description of herd management routines, grazing periods, fly and tick control measures, sanitary protocols, and other hemoparasitic or debilitating infections.

## Discussion

Although hemoplasma infections are known to be frequent [15], this study reports one of the highest detection rate published to date, with 89.4% (833/931) of positive animals. However, this study has several limits regarding sampled population, PCR interpretation, and associated factors with PCR positivity.

First, this convenience sample cannot represent a true prevalence due to uneven representation of certain herds compared with others, as well as uneven age distribution resulting from the sampling design. Sampling from animals older than 2 years may have artificially increased the detection rate. Additionally, herds with a history of clinical issues may have been more likely to be sampled during clinical activities. As a result, any generalization of the findings presented here is precluded.

Second, the comparison of results of 16S PCR and PCR specific result showed 8% of discordant results, which were not included in the second part of the study. The “16S only” could be attributed to an unidentified, although rare, hemoplasma species or differences in PCR sensitivities and the detection of low bacterial loads from several hemoplasmas, insufficient to trigger specific PCR positivity. This would represent the main hypothesis for the following reasons: (1) very few quantified positive samples of “16S only” were achieved, and these had lower bacterial loads (though not significantly due to the small number of successfully quantified “16S only”) and (2) out of the 64 samples reexamined, only 7 “16S only” tested positive again by 16S PCR, with 4 yielding interpretable results upon amplicon sequencing. However, this also raises the question of 16S PCR’s reproducibility. Although 16S PCR is supposedly one the most sensitive PCR (except for Mex), 16 samples were only specific PCR positive. Therefore, the prevalence of coinfections and factors associated with PCR positivity found in this study should be interpreted carefully. Finally, molecular detection does not necessarily reflect viable organism or active infection.

Third, the conclusion reached related to fully sampled herds (influence of age and herd on PCR positivity) should be confirmed by larger samples and more detailed management practices, especially regarding grazing periods. Moreover, some research has found significant differences in prevalence according to herds for Mw but not for CMh or coinfection [25], suggesting that it would have been more interesting to conduct such a study for each codetection type. The decision to analyze each pathogen separately was driven by the need to maintain sufficient sample sizes for stable estimates. Future work should explore multilevel models incorporating coinfection networks to better disentangle the relative contributions of herd-level and individual-level risk factors.

Codetection rates in our study were remarkably high, with 13.6% (127/931) of animals testing positive for CMh alone, 17.2% (160/931) for *Mycoplasma wenyonii* alone (all strains), and 58.6% (549/931) of codetections. These rates are concordant with global prevalence reported in other studies, which range from 0% to 56.59% for CMh, 7% to 19.5% for *M. wenyonii*, and 14.1% to 63.4% for coinfections [14, 26, 27]. However, direct comparisons remain challenging due to differences in study designs, populations sampled, and detection methods.

Although cautious interpretation is warranted given the previous exposed limitations, hemoplasma detection was associated with age in this study, peaking at around 2–3 years and then declining after 5 years, consistent with previous studies [25]. These findings align with prior research reporting lower hemoplasma prevalence in calves and heifers [25], but higher rates in cattle aged 1–3 years [28]. Similar age-related trends have been observed for other hemoparasites, such as *Anaplasma* spp. and *Theileria/Babesia* spp., which are more frequently detected in cattle under 3 years [29]. Although infected animals may act as chronic carriers [15], the observed decline in CMh and Mex prevalence with age suggests potential clearance or immune-mediated suppression over time, contrasting with previous hypothesis of lifelong infections [30]. Collectively, these observations likely reflect differences in infection capacity, persistence, immune evasion strategies, transmission dynamics, or variations in bovine exposure depending on the host’s age and physiological state.

Studies regarding location reported both consistent and highly variable hemoplasma prevalence within the same region [9, 16], suggesting that local factors, such as herd characteristics or climate, may drive these differences through vector-borne transmission dynamics. This geographic variability is evident for other hemoparasites such as *Babesia* spp., with PCR-detected prevalence ranging from 14.1% to 24.7% in China [31], while *Babesia bovis* was detected in 29% of sampled cows in another study in Brazil [32], underscoring the role of location in shaping infection. Similarly, the prevalence of *Anaplasma* spp. varied between species and locations in a study conducted in North Africa and India [33, 34].

Hemoplasma DNA has been detected in ticks, culicoides, and flies, suggesting their potential role in transmission, though direct evidence remains lacking [7, 8, 17]. The similar detection rates , frequent codetection, and close association between Mex and CMh suggest shared transmission routes, possibly involving common vectors and frequent co-exposure.

Although this remains speculative, further research is needed to clarify the link between hemoplasma prevalence and climatic conditions, including vector development timelines, hemoplasma survival within vectors, infection-to-detection delays, and alternative transmission routes (e.g., wild ruminant reservoirs, transplacental or fomite transmission). Longitudinal studies on hemoplasma loads would enhance our understanding of infection dynamics and reinfection risks.

The risk factors driving progression from hemoplasma infection to clinical disease remain unclear [15]. Clinical forms may emerge due to coinfections, immunosuppressive agents [7, 8], or stress/immunocompromise, such as post-vaccination [13]. However, some outbreaks have involved *M. wenyonii* as the sole agent [4]. Subclinical effects such as reduced milk yield or birthweight have been described [14], but remain inconsistent [15]. However, no such effect was evidenced on milk production in this study. Given the high prevalence of hemoplasma infections, its clinical and subclinical impact may be limited, raising questions about its role in the blood pathobiome or even as part of a normal “blood microbiome.” Although blood microbiome concept has not been really explored in animals, human studies suggest that blood is not a sterile environment. Bacteria such as *Chlamydiae*, *Firmicutes*, *Proteobacteria*, *Pseudomonadota*, and *Actinobacteria* have been detected in human blood. These bacteria may originate from transient translocation of commensal microbes from other body sites or have a maternal origin [35].

Transmission routes remain debated: while grazing is not consistently identified as a risk factor [28], one study linked infection to the dam’s grazing period [17]. Transplacental transmission has been demonstrated, with rates ranging from 10.2% for all hemoplasma when sampling occurs in calves before colostrum intake [8] to 42.7% for CMh and 2.7% for Mw on aborted fetuses [36]. Higher-than-assumed transplacental transmission rates may explain the presence of hemoplasmas in the bloodstream, as well as discrepancies such as low detection rates in potential vectors (7.4% in pooled houseflies) compared with high hemoplasma detection rates in cattle (50.8%) [17], or other surprising results, such as a higher CMh prevalence in buffaloes free of ticks compared with infested ones [26]. The increase in detection in animals older than 6 months [25] may also reflect the decline of maternal antibodies. Finally, further research is needed to determine the origin of hemoplasmas in cattle blood and their significance.

## Supplementary Information

**Additional file 1. Comparison of mean log₁₀-transformed CMh bacterial loads per mL of blood by detailed codetection type.** *Descriptive statistics (count, mean, standard error, minimum, first quartile, median, third quartile, and maximum) of mean log₁₀-transformed CMh bacterial loads per mL of blood by detailed codetection type*. Statistical significance of variations of mean log₁₀-transformed CMh bacterial loads per mL of blood by detailed codetection type (Wilcoxon test with Bonferroni correction applied). *Pairwise comparison of mean log₁₀-transformed CMh bacterial loads per mL of blood between different codetection groups using the Wilcoxon test with Bonferroni correction. The table lists p-values for each comparison*. Comparison of mean log₁₀-transformed Mass bacterial loads per mL of blood by detailed codetection type. *Descriptive statistics (count, mean, standard deviation, minimum, first quartile, median, third quartile, and maximum) of mean log₁₀-transformed Mass bacterial loads per mL of blood by detailed codetection type*. Statistical significance of variations of mean log₁₀-transformed Mass bacterial loads per mL of blood by detailed codetection type (Wilcoxon test with Bonferroni correction applied). *Pairwise comparison of mean log₁₀-transformed Mass bacterial loads per mL of blood between different codetection groups using the Wilcoxon test with Bonferroni correction*. *The table lists p-values for each comparison*

**Additional file 2. Comparison of mean log₁₀-transformed Mex bacterial loads per mL of blood by detailed codetection type.** *Descriptive statistics (count, mean, standard deviation, minimum, first quartile, median, third quartile, and maximum) of mean log₁₀-transformed Mex bacterial loads per mL of blood by detailed codetection type*. Statistical significance of variations of mean log₁₀-transformed Mex bacterial loads per mL of blood by detailed codetection type (Wilcoxon test with Bonferroni correction applied). *Pairwise comparison of mean log₁₀-transformed Mex bacterial loads per mL of blood between different codetection groups using the Wilcoxon test with Bonferroni correction. The table lists p-values for each comparison*.

**Additional file 3. Comparison of mean log₁₀-transformed 16S bacterial loads per mL of blood by detailed codetection type.** *Descriptive statistics (count, mean, standard error, minimum, first quartile, median, third quartile, and maximum) of 16S loads (log₁₀-transformed) per mL of blood across different codetection combinations*. Statistical significance of variations of mean log₁₀-transformed 16S bacterial loads per mL of blood by detailed codetection type (Wilcoxon test with Bonferroni correction applied) *Pairwise comparison of mean log₁₀-transformed 16S bacterial loads per mL of blood between different codetection groups using the Wilcoxon test with Bonferroni correction. The table lists p-values for each comparison*

**Additional file 4. Five dairy herds integrally sampled characteristics.** *Characteristics of five integrally sampled dairy herds, including type, breed, history, sampling date, location, herd size, milking type, milk yield, grazing system, climatic parameters (temperature, relative humidity, precipitation, wind speed), climate type, and prevalence of 16S, CMh, Mex, and Mass infections*.

**Additional file 5. Detection by hemoplasma species and strains according to herds and age categories.** Detection of hemoplasma species and strains according to herds (herd 1: n = 151, herd 2: n = 134, herd 3: n = 82, herd 4: n = 67, herd 5: n = 54) and age categories, with statistical significance indicated by * (p < 0.001), (p < 0.01), (p < 0.05) using the Fisher exact test. *Effectives are presented in additional file 11*. Age categories among each herd. Distribution of age categories (of less than 2 years, of 2 years, of 3 years, of 4 years or more) among each of the five herds (herd 1: n = 151, herd 2: n = 134, herd 3: n = 82, herd 4: n = 67, herd 5: n = 54). *Statistical significance of age category effect across PCR positivity depending on herds. Statistical significance of the effect of age category on PCR positivity (16S, CMh, Mex, Mass) across herds (1–5), with p-values calculated using Fisher’s exact test or chi-squared test*.

**Additional file 6. Milk yield per cow and per month according to each PCR status.** Milk yield per cow and per month according to each PCR status for herds 1–4 (herd 5 excluded due to unavailable lactation data). *Milk production is expressed in liters of milk per day. No significant differences were observed except for herd 1 – 16S – month 6 (Wilcoxon test, p = 0.013).*

**Additional file 7. Hemoplasma detection results and sample metadata**
Description: Database including sample identification, animal information, collection date, molecular detection results for hemoplasmas, and associated metadata.

## Data Availability

All data generated or analyzed during this study are included in this published article and its supplementary information files.
