# Supplementary material for: Investigating Mycoplasma wenyonii and Candidatus Mycoplasma haematobovis coinfection patterns in cattle from southwestern France reveals strain-specific traits
Source: Vet Res. 2026 Aug 3;57:143. doi: 10.1186/s13567-026-01821-y (PMC13430915; doi:10.1186/s13567-026-01821-y)
Supplement: Supplementary file 1 — Additional file 1. Comparison of mean log₁₀-transformed CMh bacterial loads per mL of blood by detailed codetection type. Descriptive statistics (count, mean, standard error, minimum, first quartile, median, third quartile, and maximum) of mean log₁₀-transformed CMh bacterial loads per mL of blood by detailed codetection type. Statistical significance of variations of mean log₁₀-transformed CMh bacterial loads per mL of blood by detailed codetection type (Wilcoxon test with Bonferroni correction applied). Pairwise comparison of mean log₁₀-transformed CMh bacterial loads per mL of blood between different codetection groups using the Wilcoxon test with Bonferroni correction. The table lists p-values for each comparison. Comparison of mean log₁₀-transformed Mass bacterial loads per mL of blood by detailed codetection type. Descriptive statistics (count, mean, standard deviation, minimum, first quartile, median, third quartile, and maximum) of mean log₁₀-transformed Mass bacterial loads per mL of blood by detailed codetection type. Statistical significance of variations of mean log₁₀-transformed Mass bacterial loads per mL of blood by detailed codetection type (Wilcoxon test with Bonferroni correction applied). Pairwise comparison of mean log₁₀-transformed Mass bacterial loads per mL of blood between different codetection groups using the Wilcoxon test with Bonferroni correction. The table lists p-values for each comparison [file 13567_2026_1821_MOESM1_ESM.docx]

**Table S1: Comparison of mean log₁₀-transformed CMh bacterial loads per mL of blood by detailed codetection type**

| Coinfection type -CMh | Count | Mean | SE | Min | Q1 | Med | Q3 | Max |
| --- | --- | --- | --- | --- | --- | --- | --- | --- |
| CMh_Mex | 3 | 4.88 | 0.57 | 4.48 | 4.68 | 4.88 | 5.08 | 5.28 |
| CMh_Mex_Mass | 1 | 5.46 | - | - | - | - | - | - |
| 16S_CMh | 62 | 5.1954 | 1.0964 | 3.3729 | 4.3822 | 5.0711 | 5.7980 | 8.3892 |
| 16S_CMh_Mass | 14 | 4.6176 | 0.6837 | 3.4576 | 4.1431 | 4.4741 | 5.2076 | 5.8876 |
| 16S_CMh_Mex | 211 | 5.1221 | 0.9930 | 2.8500 | 4.4890 | 4.9504 | 5.4980 | 9.5534 |
| 16S_CMh_Mex_Mass | 69 | 5.0534 | 0.8705 | 2.8525 | 4.4789 | 4.9243 | 5.6590 | 7.2345 |
| CMh_tot | 360 | 5.09 | 0.98 | 2.85 | 4.43 | 4.95 | 5.54 | 9.55 |

**Table S2: Statistical significance of variations of mean log₁₀-transformed CMh bacterial loads per mL of blood by detailed codetection type**

| Group 1 | Group2 | *p*-value |
| --- | --- | --- |
| 16S_CMh | 16S_CMh_Mass | 0.448 |
| 16S_CMh | 16S_CMh_Mex | 1.000 |
| 16S_CMh | 16S_CMh_Mex_Mass | 1.000 |
| 16S_CMh_Mass | 16S_CMh_Mex | 0.319 |
| 16S_CMh_Mass | 16S_CMh_Mex_Mass | 0.449 |
| 16S_CMh_Mex | 16S_CMh_Mex_Mass | 1.000 |

Wilcoxon test with Bonferroni correction applied

**Table S3: Comparison of mean log₁₀-transformed Mass bacterial loads per mL of blood by detailed codetection type**

| Category | Count | Mean | SD | Min | Q1 | Median | Q3 | Max |
| --- | --- | --- | --- | --- | --- | --- | --- | --- |
| Mass_CMh_Mex | 5 | 4.13 | 2.26 | 2.53 | 3.33 | 4.13 | 4.93 | 5.73 |
| 16S_Mass | 9 | 5.2432 | 0.6450 | 4.2887 | 4.9074 | 5.0253 | 5.7193 | 6.2335 |
| 16S_Mass_Mex | 10 | 4.8737 | 0.8458 | 4.0615 | 4.4827 | 4.7888 | 4.9002 | 7.1031 |
| 16S_Mass_CMh | 19 | 4.8663 | 1.4033 | 2.4136 | 4.0341 | 5.1807 | 5.8674 | 7.3328 |
| 16S_Mass_CMh_Mex | 71 | 5.1123 | 1.2669 | 1.6021 | 4.3421 | 5.3424 | 5.9070 | 7.4618 |
| Mass_toy | 114 | 5.01 | 1.23 | 1.6 | 4.27 | 5.11 | 5.8 | 7.46 |

**Table S4: Statistical significance of variations of mean log₁₀-transformed Mass bacterial loads per mL of blood by detailed codetection type**

| Group1 | Group2 | *p*-value |
| --- | --- | --- |
| 16S_Mass | 16S_Mass_CMh | 1.000 |
| 16S_Mass | 16S_Mass_CMh_Mex | 1.000 |
| 16S_Mass | 16S_Mass_Mex | 0.612 |
| 16S_Mass_CMh | 16S_Mass_CMh_Mex | 1.000 |
| 16S_Mass_CMh | 16S_Mass_Mex | 1.000 |
| 16S_Mass_CMh_Mex | 16S_Mass_Mex | 1.000 |

Wilcoxon test with Bonferroni correction applied
