# Supplementary material for: Investigating Mycoplasma wenyonii and Candidatus Mycoplasma haematobovis coinfection patterns in cattle from southwestern France reveals strain-specific traits
Source: Vet Res. 2026 Aug 3;57:143. doi: 10.1186/s13567-026-01821-y (PMC13430915; doi:10.1186/s13567-026-01821-y)
Supplement: Supplementary file 2 — Additional file 2. Comparison of mean log₁₀-transformed Mex bacterial loads per mL of blood by detailed codetection type. Descriptive statistics (count, mean, standard deviation, minimum, first quartile, median, third quartile, and maximum) of mean log₁₀-transformed Mex bacterial loads per mL of blood by detailed codetection type. Statistical significance of variations of mean log₁₀-transformed Mex bacterial loads per mL of blood by detailed codetection type (Wilcoxon test with Bonferroni correction applied). Pairwise comparison of mean log₁₀-transformed Mex bacterial loads per mL of blood between different codetection groups using the Wilcoxon test with Bonferroni correction. The table lists p-values for each comparison. [file 13567_2026_1821_MOESM2_ESM.docx]

**Table S5: Comparison of mean log₁₀-transformed Mex bacterial loads per mL of blood by detailed codetection type**

| Category | Count | Mean | SD | Min | Q1 | Median | Q3 | Max |
| --- | --- | --- | --- | --- | --- | --- | --- | --- |
| Mex | 1 | 7.73 | - | - | - | - | - | - |
| Mex-CMh | 6 | 5.35 | 0.76 | 4.55 | 4.99 | 5.43 | 5.75 | 6.08 |
| Mex-CMh-Mass | 2 | 4.89 | 1.26 | 4 | 4.44 | 4.89 | 5.33 | 5.78 |
| 16S_Mex | 61 | 5.4004 | 1.1154 | 2.6955 | 4.8976 | 5.2368 | 5.8500 | 9.1608 |
| 16S_Mex_CMh | 10 | 6.0246 | 2.3040 | 2.8007 | 5.0719 | 5.8273 | 6.4945 | 31.7451 |
| 16S_Mex_CMh_Mass | 245 | 6.1701 | 1.6938 | 3.1271 | 4.9581 | 5.8007 | 6.9930 | 11.8195 |
| 16S_Mex_Mass | 85 | 5.2273 | 0.6815 | 3.8426 | 5.1860 | 5.3495 | 5.5001 | 6.1297 |
| Mex_tot | 410 | 5.92 | 20.02 | 2.7 | 4.95 | 5.68 | 6.44 | 31.75 |

**Table S6: Statistical significance of variations of mean log₁₀-transformed Mex bacterial loads per mL of blood by detailed codetection type**

| Group 1 | Group 2 | *p*-value |
| --- | --- | --- |
| 16S_Mex | 16S_Mex_CMh | **0.026** |
| 16S_Mex | 16S_Mex_CMh_Mass | **0.042** |
| 16S_Mex | 16S_Mex_Mass | 1.000 |
| 16S_Mex_CMh | 16S_Mex_CMh_Mass | 1.000 |
| 16S_Mex_CMh | 16S_Mex_Mass | 0.574 |
| 16S_Mex_CMh_Mass | 16S_Mex_Mass | 0.587 |

Wilcoxon test with Bonferroni correction applied
