# Supplementary material for: Investigating Mycoplasma wenyonii and Candidatus Mycoplasma haematobovis coinfection patterns in cattle from southwestern France reveals strain-specific traits
Source: Vet Res. 2026 Aug 3;57:143. doi: 10.1186/s13567-026-01821-y (PMC13430915; doi:10.1186/s13567-026-01821-y)
Supplement: Supplementary file 3 — Additional file 3. Comparison of mean log₁₀-transformed 16S bacterial loads per mL of blood by detailed codetection type. Descriptive statistics (count, mean, standard error, minimum, first quartile, median, third quartile, and maximum) of 16S loads (log₁₀-transformed) per mL of blood across different codetection combinations. Statistical significance of variations of mean log₁₀-transformed 16S bacterial loads per mL of blood by detailed codetection type (Wilcoxon test with Bonferroni correction applied) Pairwise comparison of mean log₁₀-transformed 16S bacterial loads per mL of blood between different codetection groups using the Wilcoxon test with Bonferroni correction. The table lists p-values for each comparison [file 13567_2026_1821_MOESM3_ESM.docx]

**Table S7: Comparison of mean log₁₀-transformed 16S bacterial loads per mL of blood by detailed codetection type**

| Catégorie | Count | Mean | SE | Min | Q1 | Med | Q3 | Max |
| --- | --- | --- | --- | --- | --- | --- | --- | --- |
| 16S_only | 5 | 5.3527 | 0.7143 | 4.7059 | 4.8476 | 5.0835 | 5.6857 | 6.4409 |
| 16S_CMh | 67 | 5.4874 | 0.7349 | 4.1547 | 4.9300 | 5.4594 | 5.9464 | 7.1584 |
| 16S_Mex | 69 | 5.5230 | 0.7914 | 4.1608 | 4.8808 | 5.4150 | 6.0934 | 7.5888 |
| 16S_Mass | 15 | 5.8132 | 0.6739 | 4.7419 | 5.4448 | 5.7292 | 5.9638 | 7.0170 |
| 16S_CMh_Mass | 21 | 5.8249 | 0.7954 | 4.6990 | 5.2788 | 5.7482 | 6.0615 | 7.3156 |
| 16S_CMh_Mex | 273 | 5.9890 | 0.7165 | 4.3345 | 5.5823 | 5.9722 | 6.4166 | 8.7451 |
| 16S_Mex_Mass | 14 | 5.5803 | 0.6002 | 4.5127 | 5.2973 | 5.5353 | 5.9087 | 6.7160 |
| 16S_CMh_Mex_Mass | 90 | 6.2139 | 0.6476 | 4.5159 | 5.8235 | 6.2600 | 6.5499 | 7.8808 |
| 16S_tot | 554 | 5.85 | 0.76 | 3.98 | 5.3 | 5.85 | 6.37 | 8.75 |

**Table S8: Statistical significance of variations of mean log₁₀-transformed 16S bacterial loads per mL of blood by detailed codetection type**

| Group 1 | Group 2 | *p*-value |
| --- | --- | --- |
| 16S_CMh | 16S_CMh_Mass | 1.0000 |
| 16S_CMh | 16S_CMh_Mex | **0.0005** |
| 16S_CMh | 16S_CMh_Mex_Mass | **<0.0001** |
| 16S_CMh | 16S_Mass | 1.0000 |
| 16S_CMh | 16S_Mex | 1.0000 |
| 16S_CMh | 16S_Mex_Mass | 1.0000 |
| 16S_CMh | 16S_only | 1.0000 |
| 16S_CMh_Mass | 16S_CMh_Mex | 1.0000 |
| 16S_CMh_Mass | 16S_CMh_Mex_Mass | 0.4620 |
| 16S_CMh_Mass | 16S_Mass | 1.0000 |
| 16S_CMh_Mass | 16S_Mex | 1.0000 |
| 16S_CMh_Mass | 16S_Mex_Mass | 1.0000 |
| 16S_CMh_Mass | 16S_only | 1.0000 |
| 16S_CMh_Mex | 16S_CMh_Mex_Mass | 0.3470 |
| 16S_CMh_Mex | 16S_Mass | 1.0000 |
| 16S_CMh_Mex | 16S_Mex | **0.0020** |
| 16S_CMh_Mex | 16S_Mex_Mass | 0.8460 |
| 16S_CMh_Mex | 16S_only | 1.0000 |
| 16S_CMh_Mex_Mass | 16S_Mass | 0.5740 |
| 16S_CMh_Mex_Mass | 16S_Mex | **<0.0001** |
| 16S_CMh_Mex_Mass | 16S_Mex_Mass | **0.0340** |
| 16S_CMh_Mex_Mass | 16S_only | 0.6130 |
| 16S_Mass | 16S_Mex | 1.0000 |
| 16S_Mass | 16S_Mex_Mass | 1.0000 |
| 16S_Mass | 16S_only | 1.0000 |
| 16S_Mex | 16S_Mex_Mass | 1.0000 |
| 16S_Mex | 16S_only | 1.0000 |
| 16S_Mex_Mass | 16S_only | 1.0000 |

Wilcoxon test with Bonferroni correction applied
