# Supplementary material for: Investigating Mycoplasma wenyonii and Candidatus Mycoplasma haematobovis coinfection patterns in cattle from southwestern France reveals strain-specific traits
Source: Vet Res. 2026 Aug 3;57:143. doi: 10.1186/s13567-026-01821-y (PMC13430915; doi:10.1186/s13567-026-01821-y)
Supplement: Supplementary file 4 — Additional file 4. Five dairy herds integrally sampled characteristics. Characteristics of five integrally sampled dairy herds, including type, breed, history, sampling date, location, herd size, milking type, milk yield, grazing system, climatic parameters (temperature, relative humidity, precipitation, wind speed), climate type, and prevalence of 16S, CMh, Mex, and Mass infections. [file 13567_2026_1821_MOESM4_ESM.docx]

**Table S9: Characteristics of the five dairy herds integrally sampled**

| Herd | 1 | 2 | 3 | 4 | 5 |
| --- | --- | --- | --- | --- | --- |
| Type | Dairy | Dairy | Organic dairy | Dairy | Dairy |
| Breed | HF | HF | HF | HF | Montbéliarde |
| History | None | Clinical cases | None | None | None |
| Date of sampling | 03.04.2021 | 11.01.2025 | 04.01.2025 | 19.09.2023 | 19.09.2023 |
| Department | Haute-Garonne | Cantal | Cantal | Ardèche | Ardèche |
| Number of bovines | 151 | 134 | 82 | 67 | 54 |
| Milking type | AMS | AMS | Milking machine | AMS | Milking machine |
| Milk yield/cow/305 DIM | 12047 L | 14553 L | 7540 L | 8906 L | 8300 L |
| Grazing system | No grazing | March to November:  heifers of 10-24 months old | March to November:  dairy cows and heifers > 6 months old | March to December:  dry cows and heifers > 6 months old | March to July:  dairy cows  March to November:  Heifers > 6 months old |
| T (°C) | 14.41 °C | 10.86 °C | 10.56 °C | 7.81 °C | 12.39 °C |
| RH (%) | 70.88% | 80.51% | 81.26% | 72.6% | 66.01% |
| P (mm) | 3.64 mm | 5.24 mm | 6.59 mm | 5.88 mm | 3.26 mm |
| WS (m/s) | 14.67 m/s | 10.67 m/s | 10.42 m/s | 7.65 m/s | 12.03 m/s |
| Climate type | Mediterranean | Semi-oceanic | Semi-oceanic | Mediterranean | Mediterranean |
| 16S prevalence | 87.4% | 94% | 97.6% | 79.1% | 79.2% |
| CMh prevalence | 76.2% | 88.8% | 86.6% | 44.8% | 61.1% |
| Mex prevalence | 72.2% | 77.6% | 67.1% | 70.1% | 48.1% |
| Mass prevalence | 16.6% | 23.9% | 39% | 14.9% | 27.8% |

*Herd 2 and Herd 3 are 8km apart; Herd 4 and Herd 5 are 22 km apart; AMS: Automatic Milking System; DIM: days in milk; coinfection patterns are represented on figure 2. Clinical cases were compatible with hemoplasma infection; T: annual mean daily temperature (°C); RH: annual mean daily relative humidity (%); WS: annual mean daily wind speed (m/s); P: annual mean daily precipitation (mm); dry cow: depending on season, pasture time/year last 2 to 3 months maximum*
