# Supplementary material for: Investigating Mycoplasma wenyonii and Candidatus Mycoplasma haematobovis coinfection patterns in cattle from southwestern France reveals strain-specific traits
Source: Vet Res. 2026 Aug 3;57:143. doi: 10.1186/s13567-026-01821-y (PMC13430915; doi:10.1186/s13567-026-01821-y)
Supplement: Supplementary file 5 — Additional file 5. Detection by hemoplasma species and strains according to herds and age categories. Detection of hemoplasma species and strains according to herds (herd 1: n = 151, herd 2: n = 134, herd 3: n = 82, herd 4: n = 67, herd 5: n = 54) and age categories, with statistical significance indicated by * (p < 0.001), (p < 0.01), (p < 0.05) using the Fisher exact test. Effectives are presented in additional file 11. Age categories among each herd. Distribution of age categories (of less than 2 years, of 2 years, of 3 years, of 4 years or more) among each of the five herds (herd 1: n = 151, herd 2: n = 134, herd 3: n = 82, herd 4: n = 67, herd 5: n = 54). Statistical significance of age category effect across PCR positivity depending on herds. Statistical significance of the effect of age category on PCR positivity (16S, CMh, Mex, Mass) across herds (1–5), with p-values calculated using Fisher’s exact test or chi-squared test. [file 13567_2026_1821_MOESM5_ESM.docx]

**Figure S1: Detection, by hemoplasma species and strains, according to herds and age categories**


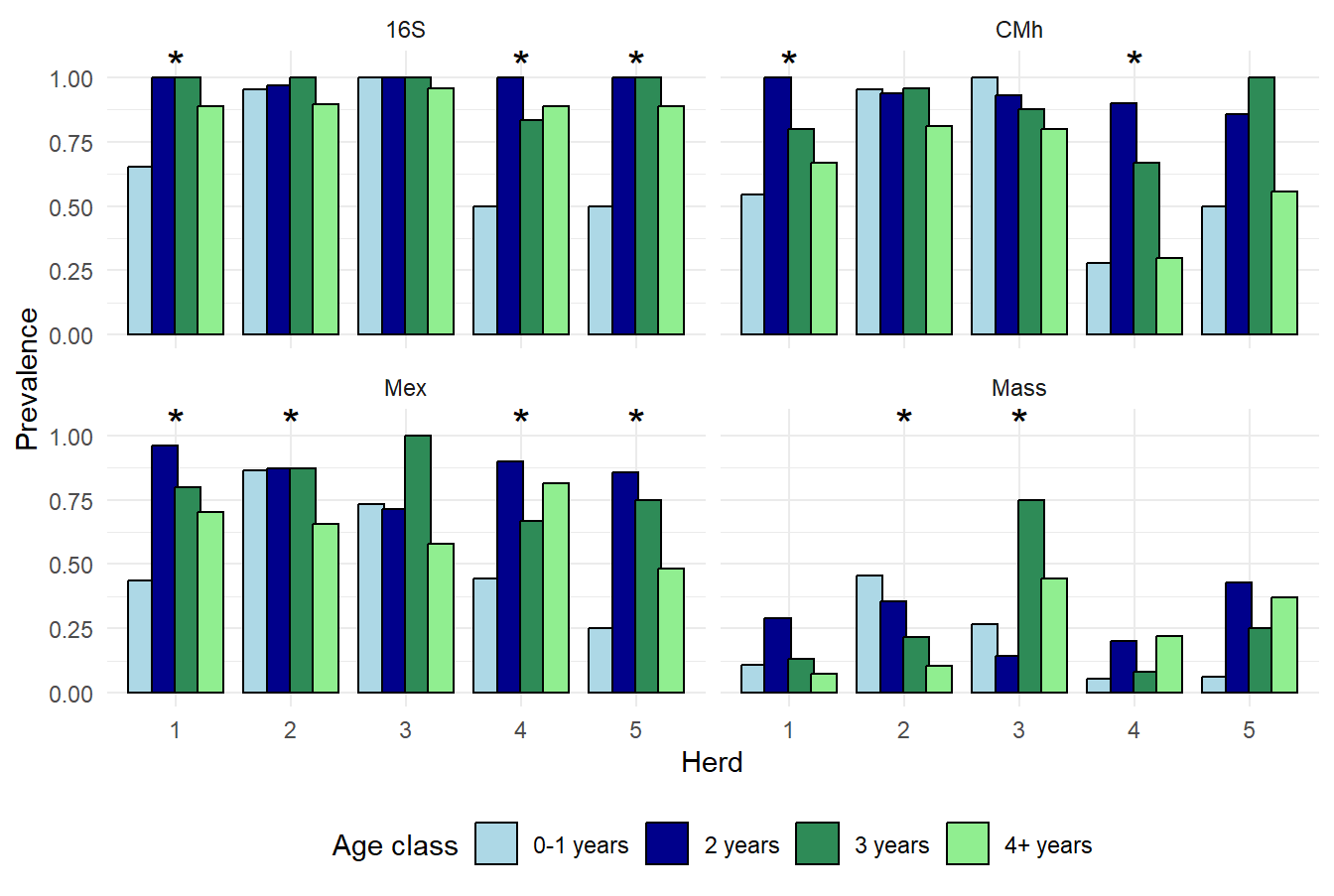


*Herd 1: n=151 animals; Herd 2: n=134 animals, Herd 3: n=82 animals; Herd 4: n=67 animals; Herd 5: n=54 animals; statistical significance: *** p <0.001, ** p <0.01, * p <0.05, Fisher exact test; effectives are presented in Table S10*

**Table S10: Age categories among each herd**

| Age category | Herd 1 | Herd 2 | Herd 3 | Herd 4 | Herd 5 |
| --- | --- | --- | --- | --- | --- |
| Of less than 2 years | 46 | 22 | 15 | 18 | 16 |
| Of 2 years | 48 | 31 | 14 | 10 | 7 |
| Of 3 years | 30 | 23 | 8 | 12 | 4 |
| Of 4 years and more | 27 | 58 | 45 | 27 | 27 |
| Total | 151 | 134 | 82 | 67 | 54 |

**Table S11: Statistical significance of age category effect across PCR positivity depending on herds**

| PCR | Herd | Test | *p*-value |
| --- | --- | --- | --- |
| 16S | 1 | Fisher | **1.40 × 10⁻⁷** |
|  | 2 | Fisher | 3.92 × 10⁻¹ |
|  | 3 | Fisher | 1.00 × 10⁰ |
|  | 4 | Fisher | **4.17 × 10⁻³** |
|  | 5 | Fisher | **9.21 × 10⁻³** |
| CMh | 1 | Chi² | **2.63 × 10⁻⁶** |
|  | 2 | Fisher | 1.56 × 10⁻¹ |
|  | 3 | Fisher | 2.35 × 10⁻¹ |
|  | 4 | Fisher | **1.42 × 10⁻³** |
|  | 5 | Fisher | 1.57 × 10⁻¹ |
| Mex | 1 | Chi² | **2.91 × 10⁻⁷** |
|  | 2 | Fisher | **4.56 × 10⁻²** |
|  | 3 | Fisher | 1.07 × 10⁻¹ |
|  | 4 | Fisher | **2.95 × 10⁻²** |
|  | 5 | Fisher | **3.24 × 10⁻²** |
| Mass | 1 | Fisher | 5.14 × 10⁻² |
|  | 2 | Chi² | **3.14 × 10⁻³** |
|  | 3 | Fisher | **2.41 × 10⁻²** |
|  | 4 | Fisher | 4.06 × 10⁻¹ |
|  | 5 | Fisher | 7.66 × 10⁻² |
