# Supplementary material for: Investigating Mycoplasma wenyonii and Candidatus Mycoplasma haematobovis coinfection patterns in cattle from southwestern France reveals strain-specific traits
Source: Vet Res. 2026 Aug 3;57:143. doi: 10.1186/s13567-026-01821-y (PMC13430915; doi:10.1186/s13567-026-01821-y)
Supplement: Supplementary file 6 — Additional file 6. Milk yield per cow and per month according to each PCR status. Milk yield per cow and per month according to each PCR status for herds 1–4 (herd 5 excluded due to unavailable lactation data). Milk production is expressed in liters of milk per day. No significant differences were observed except for herd 1 – 16S – month 6 (Wilcoxon test, p = 0.013). [file 13567_2026_1821_MOESM6_ESM.docx]

**Figure S2: Milk yield per cow and per month according to each PCR status**


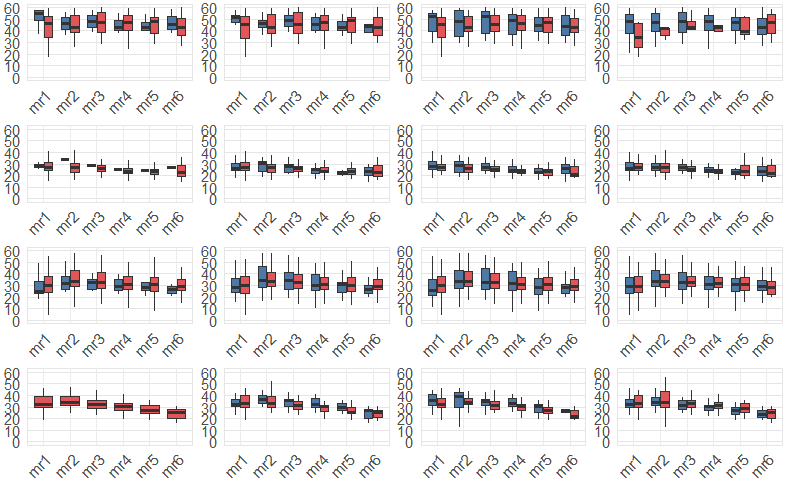


Herd 1

Herd 4

Herd 3

Herd 2

Mass

Mex

CMh

16S

*Herd 5 was not included due to unavailable data during lactation. Milk production is expressed in liter of milk per day. mrx: month of lactation number x; red: PCR positive; blue: PCR negative. There was no significant difference except for Herd 1 – Hemo – month 6 (Wilcoxon test, p = 0.013).*
